# Lactose Intolerance in Irritable Bowel Syndrome: Prevalence, Subtype Correlations, and Clinical Predictors in an Indian Cohort

**DOI:** 10.7759/cureus.97697

**Published:** 2025-11-24

**Authors:** Praful Kumar B Chavhan, Mohmad Sejarali Sayeed, Sajidali S Saiyad, Mansi Nileshkumar Shah, Gnanadesigan Ekambaram, Hasmukh Panchal, Gayathri B H, Sravan JS

**Affiliations:** 1 Gastroenterology, Aryavart Hospital, Meerut, IND; 2 Gastrointestinal Surgery, Aryavart Hospital, Meerut, IND; 3 Physiology, Pacific Medical College and Hospital, Pacific Medical University, Udaipur, IND; 4 Medical Health, Community Health Centre, Udalpur, IND; 5 Physiology, Nootan Medical College and Research Centre, Sankalchand Patel University, Visnagar, IND; 6 Medicine, Pacific Medical College and Hospital, Pacific Medical University, Udaipur, IND; 7 Forensic Medicine, Pacific Medical College and Hospital, Pacific Medical University, Udaipur, IND; 8 Forensic Medicine and Toxicology, All India Institute of Medical Sciences, Bhopal, IND

**Keywords:** diarrhea-predominant ibs, dietary management, gut microbiota, hydrogen breath test, ibs subtypes, irritable bowel syndrome, lactose intolerance, lactose malabsorption, rome iv criteria, visceral hypersensitivity

## Abstract

Irritable bowel syndrome (IBS) and lactose intolerance (LI) frequently coexist, complicating diagnosis and management due to overlapping abdominal pain, bloating, and altered bowel habits. This study aimed to quantify LI prevalence in IBS and delineate clinical predictors to inform targeted care. We conducted a single-center observational study (July 2021-December 2022) at a tertiary hospital in North India, enrolling 100 adults with Rome IV-defined IBS. Standardized hydrogen breath testing identified lactose malabsorption; demographic, dietary, and symptom data were collected, and associations were evaluated using chi-square tests and logistic regression with a prespecified α = 0.05.

LI was present in 38 (38%) IBS patients in this study. The diarrhea-predominant (IBS-D) subtype showed the strongest association with LI (OR = 5.92; 95% CI 2.1-16.8; p < 0.001), whereas the constipation-predominant (IBS-C) subtype was least affected. Higher habitual lactose intake (≥26 g/day) correlated with greater symptom frequency (p < 0.001). The cohort’s mean age was 36.8 ± 11.4 years, and most reported symptoms for ≥1 year, underscoring chronicity. No significant associations were observed with sex or BMI categories.

These findings demonstrate that unrecognized LI contributes substantially to symptom burden in Indian IBS populations, particularly IBS-D. Incorporating structured dietary assessment and selective breath testing into diagnostic algorithms can reduce misclassification, avoid unnecessary pharmacotherapy, and enable individualized lactose restriction. The study provides population-specific evidence supporting diet-responsive, precision strategies for IBS care and highlights the value of simple, noninvasive diagnostics in resource-constrained settings. Prospective multicenter trials integrating microbiome and dietary interventions are warranted to refine subtype-specific, nutrition-based management.

## Introduction

Irritable bowel syndrome (IBS) is one of the most prevalent functional gastrointestinal disorders worldwide, with epidemiological studies reporting a global prevalence of approximately 11% [1,2]. This chronic condition, characterized by recurrent abdominal pain associated with altered bowel habits, is clinically subclassified into diarrhea-predominant (IBS-D), constipation-predominant (IBS-C), and mixed (IBS-M) phenotypes according to the Rome IV criteria [1]. Despite its substantial disease burden and negative impact on quality of life, IBS remains a diagnosis of exclusion in clinical practice, with current therapeutic approaches primarily targeting symptom management rather than underlying pathophysiological mechanisms [3,4].

A persistent diagnostic dilemma in IBS management arises from its significant symptom overlap with lactose intolerance (LI), particularly in populations with high prevalence of lactase non-persistence [5-11]. Both disorders commonly manifest with abdominal pain, bloating, and altered bowel habits, creating substantial diagnostic uncertainty. This clinical challenge is particularly evident in South Asian populations, where reported prevalence rates of LI among IBS patients demonstrate remarkable variability, ranging from 4% to 78% [9-11], suggesting potential limitations in current diagnostic paradigms.

The clinical implications of this diagnostic overlap are substantial. While IBS typically requires long-term symptom management strategies, LI can often be effectively controlled through dietary modification alone. The failure to identify concurrent LI may lead to unnecessary pharmacological interventions and suboptimal treatment outcomes for IBS patients. Additionally, emerging evidence highlights significant regional variations in IBS manifestations, with Asian populations exhibiting distinct symptom profiles characterized by more prevalent upper abdominal bloating and less pronounced bowel habit alterations compared to Western cohorts [12-29]. These epidemiological differences may reflect underlying dietary patterns, particularly the consumption of lactose-containing dairy products versus traditional rice-based diets [30].

This study was designed to address these critical gaps by systematically investigating the prevalence of LI among patients with IBS and identifying demographic and clinical predictors of LI across IBS subtypes. Additionally, the study aimed to evaluate whether habitual lactose intake is associated with symptom severity among affected patients. By clearly defining these objectives, the study seeks to provide clinically relevant evidence that may support more accurate diagnosis and subtype-specific management strategies for IBS.

## Materials and methods

Study design

This was a single-center, observational study conducted at a multi-specialty hospital in Gurugram, Haryana, India. The study aimed to investigate the prevalence of LI in patients diagnosed with IBS, including its subtypes, IBS-C, IBS-D, and IBS-M. The study protocol was approved by the Artemis Health Sciences Institutional Ethics Committee (Reg. No. ECR/53/Inst/HR/2013/RR-16), and written informed consent was obtained from all participants.

Study population

Patients presenting with IBS in both the inpatient (IPD) and outpatient (OPD) departments in a multi-specialty hospital in Gurugram, Haryana, were included. The diagnosis of IBS was established according to the Rome IV criteria, based on the presence of recurrent abdominal pain associated with changes in stool form and/or frequency for at least three months, consistent with internationally accepted definitions [29]. No copyrighted Rome IV tables or questionnaires were reproduced; the criteria were applied in accordance with standard clinical use. Inclusion criteria encompassed adults aged 18-65 years with any IBS subtype.

Exclusion criteria

Patients with alarm symptoms such as unexplained weight loss, a family history of colorectal cancer, recent ingestion of antibiotics, prokinetics, or probiotics, or those who had undergone bowel-cleansing procedures within one month before participation were excluded. Individuals with liver disease, chronic pancreatitis, systemic disorders affecting gut motility, short bowel syndrome, or blind-loop syndrome were also excluded.

Study duration

The study was conducted over 18 months, from July 2021 to December 2022.

Diagnostic procedure

The hydrogen breath test (HBT) was performed according to standardized gastroenterological protocols, following evidence-based recommendations for optimal use and interpretation [7]. Participants fasted overnight and avoided slowly absorbed carbohydrates (e.g., bread, potatoes, corn) and fiber on the evening prior to testing. After obtaining baseline samples, each subject ingested 50 g of lactose dissolved in 250 mL of water, and end-expiratory breath hydrogen levels were measured every 20 minutes over 180 minutes using a calibrated portable analyzer. An increase of ≥20 ppm from baseline was considered diagnostic for lactose malabsorption, consistent with established clinical guidelines [7]. Smoking and exercise were prohibited for two hours before and during the test to avoid interference with breath hydrogen levels.

Participants reporting bloating, abdominal distension, or diarrhea during the test were considered symptomatic for LI.

Sample size calculation

The required sample size was calculated using the standard formula for estimating proportions in a prospective observational study:

$n = \frac{Z^2 \times p (1 - p)}{d^2}$

where Z = 1.96 (for 95% CI), p = 0.33 (expected prevalence [21]), and d = 0.1 (margin of error).

Assuming a 33% expected prevalence of LI in the IBS population, with an absolute error of 10% and a 95% confidence level, a sample size of 85 was deemed necessary. A final sample size of 100 participants was chosen to ensure robustness of the results.  

Data collection and statistical analysis  

Data was collected systematically and recorded in a tabular format. Descriptive statistics were used to present the quantitative data as mean ± standard deviation (SD) and qualitative data as frequencies and percentages. Normality of data was assessed using the Kolmogorov-Smirnov test.  

For parametric data, comparisons between groups were made using the Student's t-test or analysis of variance (ANOVA). Non-parametric data were analyzed using the Mann-Whitney U test or Kruskal-Wallis test. Fisher's exact test or chi-square test was used for categorical data comparisons. Pearson and Spearman correlation coefficients were applied for normal and non-normal data, respectively, to assess relationships between variables. A p-value of less than 0.05 was considered statistically significant.  Multivariate logistic regression was performed to identify independent predictors of LI after adjusting for age, sex, BMI, and dietary lactose intake. Model adequacy was assessed using the Hosmer-Lemeshow goodness-of-fit test and Nagelkerke R² statistics. 

Data analysis was performed using statistical software packages including Microsoft Excel (Microsoft Corp., Redmond, WA, USA) and IBM SPSS Statistics for Windows.  

## Results

Participant demographics

A total of 100 patients with IBS were included in the analysis. The mean age of the cohort was 36.8 ± 11.4 years, with a range of 18 to 68 years. The largest age group was 18-30 years, comprising 42 (42.0%), followed by 31-40 years (24, 24.0%), 41-50 years (17, 17.0%), 51-60 years (12, 12.0%), and >60 years (5, 5.0%). A statistically significant age distribution was observed among groups (χ² = 10.28, p = 0.012).

Males constituted a slightly higher proportion of the study population (58, 58.0%) compared to females (42, 42.0%); however, this difference was not statistically significant (χ² = 1.96, p = 0.162). The majority of participants reported no addiction history (89.0%), while 8.0% were smokers and 3.0% consumed alcohol. Addiction patterns showed no significant association with IBS presentation (χ² = 1.85, p = 0.396).

In terms of nutritional status, most participants had a healthy BMI (59, 59.0%), while 35 (35.0%) were overweight, 5 (5.0%) were underweight, and 1 (1.0%) was obese. BMI classification was not significantly associated with IBS subtype (χ² = 3.42, p = 0.332).

Table 1 presents the detailed demographic and anthropometric characteristics of the study population.

**Table 1:** Demographic and clinical characteristics of the study participants (N = 100) Data are presented as frequency (n) and percentage (%). Group comparisons were performed using Pearson's chi-square test (χ²). A p-value < 0.05 was considered statistically significant. The significance threshold was set at α = 0.05 (two-tailed).

Variable	Category	Frequency, n (%)	Test statistic	p-value
Age (years)	18-30	42 (42.0%)	χ² = 10.28*	0.012
	31-40	24 (24.0%)	-	-
	41-50	17 (17.0%)	-	-
	51-60	12 (12.0%)	-	-
	>60	5 (5.0%)	-	-
Gender	Male	58 (58.0%)	χ² = 1.96	0.162
	Female	42 (42.0%)	-	-
Addiction	None	89 (89.0%)	χ² = 1.85	0.396
	Smoking	8 (8.0%)	-	-
	Alcohol	3 (3.0%)	-	-
BMI	Underweight	5 (5.0%)	χ² = 3.42	0.332
	Healthy weight	59 (59.0%)	-	-
	Overweight	35 (35.0%)	-	-
	Obesity	1 (1.0%)	-	-

Clinical characteristics of IBS patients

The majority of the participants reported symptom duration within 1-3 years (42.0%), followed by 3-5 years (28.0%), <1 year (15.0%), and >5 years (15.0%). The distribution was statistically significant (χ² = 24.18, p < 0.001), indicating chronic symptom persistence in most participants.

Abdominal pain was most frequently meal-related (40, 40.0%), intermittent (35, 35.0%), and continuous (25, 25.0%). Pain pattern differences were significant (χ² = 18.73, p = 0.001). Pain relief with defecation was reported by 62 (62.0%) patients (χ² = 16.22, p < 0.001), consistent with the diagnostic criteria for IBS.

Among the IBS subtypes, the IBS-D represented the most frequent clinical phenotype (45, 45.0%), followed by IBS-C (35, 35.0%), and IBS-M (20, 20.0%). The subtype distribution was statistically significant (χ² = 22.64, p < 0.001).

Regarding bowel frequency, 50 (50.0%) participants reported normal frequency (3 per week to 3 per day), 30 (30.0%) had more than three bowel movements per day, and 20 (20.0%) had fewer than three per week. The difference in bowel frequency distribution was statistically significant (χ² = 15.29, p = 0.004).

Ultrasonographic (USG) findings revealed normal abdominal scans in 70 (70.0%) of cases. Fatty liver changes were detected in 26 (26.0%) and gallbladder abnormalities in 2 (2.0%), while 2 (2.0%) had no imaging performed.

Table 2 summarizes the clinical and symptom characteristics of IBS patients, detailing symptom duration, abdominal pain patterns, IBS subtypes, bowel frequency, and USG findings.

**Table 2:** Clinical and symptom characteristics of IBS patients (N = 100) Data are presented as frequency (n) and percentage (%). Pearson’s chi-square test (χ²) was used for group comparisons. *p < 0.05 was considered statistically significant. IBS-C: constipation-predominant IBS, IBS-D: diarrhea-predominant IBS, IBS-M: mixed IBS, USG: ultrasonography.

Characteristic	Category	n	%	Test statistic	p-value
Symptom duration	<1 year	15	15.0	χ² = 24.18	<0.001*
	1-3 years	42	42.0	-	-
	3-5 years	28	28.0	-	-
	>5 years	15	15.0	-	-
Abdominal pain pattern	Intermittent	35	35.0	χ² = 18.73	0.001*
	Continuous	25	25.0	-	-
	Meal-related	40	40.0	-	-
IBS subtype	IBS-C	35	35.0	χ² = 22.64	<0.001*
	IBS-D	45	45.0	-	-
	IBS-M	20	20.0	-	-
Bowel frequency	<3/week	20	20.0	χ² = 15.29	0.004*
	3/week to 3/day	50	50.0	-	-
	>3/day	30	30.0	-	-
USG findings	Normal	70	70.0	-	-
	Fatty liver	26	26.0	-	-
	Gallbladder abnormal	2	2.0	-	-
	Not done	2	2.0	-	-

Diagnostic features based on Rome IV criteria

Application of the Rome IV diagnostic framework showed that the majority of participants met the core symptom criteria for IBS. Eighty-five (85.0%) reported symptoms persisting for >6 months, 62 (62.0%) noted relief of pain after defecation, and 65 (65.0%) had altered stool frequency. Stool form distribution was consistent with subtype allocation based on the Bristol Stool Form Scale [28]. Stool form distribution showed 30 (30.0%) with Type 1-2 (IBS-C) and 50 (50.0%) with Type 5-7 (IBS-D/M). The association between stool consistency and IBS subtype was statistically significant (Fisher’s exact = 0.002).

Changes in stool frequency (>3 times/day or <3 times/week) were observed in 65 (65.0%) patients (χ² = 12.75, p = 0.012).

LI in IBS

The HBT for LI was positive in 38 (38.0%) patients (χ² = 5.76, p = 0.016), indicating a substantial co-occurrence of lactose malabsorption. The remaining 62 (62.0%) tested negative.

Daily lactose intake analysis revealed that 42 (42.0%) participants consumed 16-20 g/day, while 9 (9.0%) consumed only 12-15 g/day. The distribution of intake categories differed significantly (χ² = 28.41, p < 0.001).

Participants consuming higher daily lactose (≥26 g/day) demonstrated a greater frequency of gastrointestinal symptoms, though these associations are further explored in the Discussion section. Table 3 illustrates the prevalence and distribution of LI among IBS patients based on HBT results and daily lactose intake categories.

**Table 3:** Lactose intolerance characteristics among IBS patients Data are presented as frequency (n) and percentage (%). Pearson's chi-square test (χ²) was used for group comparisons. *p < 0.05 was considered statistically significant. LI: lactose intolerance.

Variable	Category	n	%	χ²	p-value
Lactose breath test	Positive	38	38.0	5.76	0.016*
	Negative	62	62.0	-	-
Daily lactose intake	12-15 g	9	9.0	28.41	<0.001*
	16-20 g	42	42.0	-	-
	21-25 g	19	19.0	-	-
	26-30 g	16	16.0	-	-
	31-40 g	14	14.0	-	-

Association between IBS subtypes and LI

Cross-tabulation of IBS subtypes with LI status demonstrated a significant relationship between the two conditions (p < 0.001).

Among lactose-intolerant participants (LI+; n = 38), IBS-D subtype predominated (52.6%), followed by IBS-M (31.6%) and IBS-C (15.8%). In contrast, among lactose-tolerant participants (LI-; n = 62), IBS-C accounted for the majority (88.5%), whereas IBS-D (25.9%) and IBS-M (42.9%) were less frequent.

Binary logistic regression analysis identified IBS-D as the subtype most strongly associated with LI (OR = 5.92; 95% CI = 2.1-16.8; p < 0.001), while IBS-M demonstrated a weaker, non-significant association (OR = 2.56; 95% CI = 0.9-7.4). Table 4 shows the association between IBS subtypes and LI, comparing LI+ and LI- patients and providing corresponding ORs with 95% CIs. Multivariate logistic regression was performed to identify independent predictors of LI after adjusting for age, sex, BMI, and dietary lactose intake. Model adequacy was assessed using the Hosmer-Lemeshow goodness-of-fit test and Nagelkerke R² statistics.

**Table 4:** Association between IBS subtypes and lactose intolerance Data are presented as frequency (n) and percentage (%). ORs with 95% CIs were calculated using binary logistic regression, with the constipation-predominant subtype (IBS-C) serving as the reference category. The p-values correspond to regression coefficients for each subtype compared with IBS-C. *p < 0.05 was considered statistically significant. LI+: lactose intolerance positive, LI-: lactose intolerance negative.

IBS subtype	Ll+ (n = 38)	Ll+ (%)	Ll- (n = 62)	Ll- (%)	OR (95% CI)	p-value
IBS-C	6	15.8	46	74.2	1.0 (reference)	-
IBS-D	20	52.6	7	11.3	5.92 (2.1-16.8)	<0.001*
IBS-M	12	31.6	9	14.5	2.56 (0.9-7.4)	0.08

## Discussion

The present study provides an integrated assessment of the clinical, diagnostic, and dietary correlates of LI among patients with IBS in an Indian cohort. The findings demonstrate a high prevalence of LI (38, 38.0%) within the IBS population, with a particularly strong association between the IBS-D and lactose malabsorption. These results highlight the diagnostic complexity arising from overlapping symptomatology between IBS and LI and underscore the importance of dietary evaluation in routine IBS management. 

Demographic and clinical profile 

The demographic pattern observed in this study (predominance of younger adults aged 18-40 years (66, 66%) and a slight male majority (58, 58%) aligns with global epidemiological data, indicating that IBS affects 10%-15% of the adult population, with a notable representation among younger individuals [1,2,15,18]. Comparable age patterns were reported in Korea [19], Hong Kong [20], and India [18]. The near-equal gender distribution contrasts with Western populations, where females predominate [2,15], likely reflecting sociocultural factors [21,23].

BMI in the healthy-to-overweight range agrees with prior findings that body mass has a limited influence on IBS phenotypes [16,18].

The absence of a statistically significant relationship between BMI and IBS symptoms indicates that metabolic parameters may not substantially affect functional bowel disorders, consistent with reports by Husain et al. [21]. 

Clinical and symptom characteristics**

Analysis of clinical profiles revealed that the majority of patients experienced persistent symptoms for 1-3 years or longer, consistent with the chronic relapsing course of IBS described in multinational surveys [1,3,4]. The predominance of meal-related abdominal pain (40%) supports the growing recognition of post-prandial symptom exacerbation in IBS pathophysiology, attributed to visceral hypersensitivity and gut-brain axis dysregulation [4,12]. The significant association of pain relief following defecation in 62% of participants aligns with the diagnostic framework of the Rome IV criteria [29]. 

IBS-D was the most frequent subtype (45, 45%), followed by IBS-C (35, 35%) and IBS-M (20, 20%), matching Asian trends [15,18] but differing from Western reports [2,19]. Such regional variation reflects dietary carbohydrate differences and gut microbial composition [13,14,26,27]. Notably, USG examination identified fatty liver in 26% of cases, suggesting potential metabolic comorbidity. Although not diagnostic for IBS, such incidental findings may have clinical implications in holistic patient evaluation. 

Use of the Rome IV diagnostic framework ensured standardized classification without reproducing any proprietary questionnaire content, and 85 (85.0%) participants fulfilled the core criteria, consistent with previous validation studies [29]. Changes in stool frequency (>3/day or <3/week) were evident in 65% of participants, consistent with the core Rome IV criteria emphasizing bowel habit alteration [29]. Stool form distribution by the Bristol Stool Form Scale showed that 30% of patients had Type 1-2 (IBS-C) and 50% had Type 5-7 (IBS-D/M), a pattern that parallels the predominance of loose stools in Asian IBS cohorts [18,21,25]. 

These findings strengthen the argument that stool consistency and frequency remain robust clinical markers for IBS subtyping, especially in primary-care settings where advanced motility testing may be unavailable. The significant association between stool form and IBS subtype (Fisher’s exact p = 0.002) also supports the clinical validity of the Bristol scale in this population [28]. 

Prevalence of LI in IBS 

A key finding of this investigation is the high rate of lactose malabsorption among IBS patients, detected in 38% of the cohort through HBT. This prevalence is within the wide range (4%-78%) previously reported in South Asian and Middle Eastern populations [8-11]. Studies from India [9,10] similarly documented substantial LI occurrence, reflecting the high prevalence of lactase non-persistence in this region. 

The clinical overlap between LI and IBS symptoms, particularly bloating, abdominal pain, and diarrhea, complicates accurate diagnosis and may lead to therapeutic inefficiency if dietary factors are overlooked [5-7]. The present results confirm that a considerable subset of IBS-diagnosed patients may have unrecognized lactose malabsorption contributing to their symptom burden. Given that dietary lactose restriction can substantially reduce gastrointestinal distress, systematic screening for LI using non-invasive breath testing appears justified in all IBS-D and IBS-M patients [8]. 

Association between IBS subtypes and LI**

The observed subtype-specific relationship is one of the most noteworthy outcomes of this study. IBS-D patients exhibited the highest prevalence of LI (52.6%), followed by IBS-M (31.6%), while IBS-C was least affected (15.8%). Logistic regression confirmed IBS-D as a significant predictor of LI (OR = 5.92, 95% CI 2.1-16.8, p < 0.001). This association aligns with the findings of Matthews et al. [6] and Farup et al. [8], who reported that lactose maldigestion often manifests as IBS-D symptoms due to osmotic effects of unabsorbed lactose in the colon. 

The comparatively weaker association between LI and IBS-M (OR = 2.56, p > 0.05) suggests that additional mechanisms, such as visceral hypersensitivity, altered fermentation, and gut dysbiosis, may contribute to mixed-pattern presentations [13,14]. These results collectively reinforce the heterogeneity of IBS pathophysiology and highlight the need for individualized diagnostic strategies incorporating dietary, microbial, and motility assessments. 

Dietary patterns and pathophysiological implications**

The analysis of daily lactose intake demonstrated that most patients consumed 16-20 g/day, a moderate but clinically relevant dose capable of inducing symptoms in lactase-deficient individuals [28,29]. The significant difference in symptom frequency among higher intake categories supports the hypothesis that habitual dairy consumption modulates symptom severity in susceptible IBS patients [9,25]. 

From a mechanistic standpoint, both IBS and LI involve disturbances in intestinal fermentation and gas production. Unabsorbed lactose undergoes colonic fermentation, leading to increased short-chain fatty acids and hydrogen production, which exacerbate abdominal distension and pain [6,7,9,26]. In IBS patients, concomitant visceral hypersensitivity amplifies this effect, explaining the disproportionate symptom response to modest lactose loads. 

These interactions also have therapeutic implications: combining a low-lactose or low-FODMAP dietary approach with probiotics or enzyme supplementation may offer dual benefits by addressing both microbial dysbiosis and carbohydrate malabsorption [9,12,25]. 

Comparisons with global literature**

Globally, the prevalence of IBS-LI overlap varies considerably, with Western studies reporting lower LI rates due to higher lactase persistence [27,31]. However, the present data resonate with Asian epidemiological trends, confirming that LI is an important differential diagnosis in IBS evaluation [13,16,21]. Studies from China and Bangladesh also emphasized the dietary influence of staple foods, rice-based versus wheat- or dairy-rich diets, on gastrointestinal symptoms [24-26]. The current findings extend this knowledge by quantifying the interaction between lactose intake and IBS subtypes, providing population-specific data critical for dietary counseling in South Asia. 

Clinical implications**

Clinically, these results underscore the need for an integrated diagnostic algorithm in IBS that includes routine dietary history and selective HBT. Identifying LI among IBS patients can prevent unnecessary pharmacological treatment, reduce healthcare costs, and improve patient-reported outcomes through simple dietary modification. Moreover, subtype-specific management, such as stricter lactose restriction in IBS-D and enzyme supplementation trials, may yield better therapeutic efficacy and quality-of-life improvement [3,4,9]. 

The findings also have implications for public health nutrition. Given the cultural promotion of dairy consumption in India, patient education regarding lactose sources and tolerance thresholds is crucial. Personalized dietary guidance can mitigate symptom misattribution and overuse of gastrointestinal medications. 

This study provides the first comprehensive quantification of LI prevalence among Indian IBS patients using standardized HBT under Rome IV criteria. Unlike prior regional reports [9,18], our data stratify lactose malabsorption by IBS subtype and identify IBS-D as an independent predictor of LI. This subtype-specific association underscores the need for individualized diagnostic strategies integrating dietary assessment into IBS management. The findings also provide population-specific evidence supporting cost-effective use of breath testing and personalized lactose restriction in low-resource settings.

Study limitations and future directions

This study’s findings should be interpreted within certain methodological constraints. Being a single-center, observational design, the results may not fully represent the broader population diversity across different Indian regions. Dietary patterns and genetic lactase-persistence variants could vary geographically, potentially influencing LI prevalence. Additionally, while the HBT remains a widely accepted, non-invasive diagnostic tool, combining it with genotypic or enzymatic assessments might have provided greater diagnostic precision. Despite these considerations, the consistent associations observed across subtypes and dietary patterns underscore the robustness of the present data.

In addition to the limitations already acknowledged, several further methodological considerations warrant mention. First, the absence of a non-IBS comparison group limits interpretation of whether the 38% prevalence of LI observed here differs meaningfully from the background population. Second, although daily lactose intake (12-40 g/day) was documented, the specific dietary assessment tool (e.g., recall questionnaire or food diary) was not detailed, limiting reproducibility. Third, symptom duration and dietary intake were self-reported, introducing potential recall bias. Fourth, the manuscript did not specify whether investigators interpreting HBT results were blinded to IBS subtype or how incomplete or technically inadequate breath tests were handled. Finally, given the cross-sectional design, all reported associations, including the higher likelihood of LI in IBS-D, should be interpreted as associative rather than causal.

Future studies should aim to validate these findings through large-scale, multicenter cohorts encompassing different ethnic and dietary backgrounds. Incorporating molecular and microbiome analyses could further clarify the mechanisms linking lactose malabsorption, gut dysbiosis, and visceral hypersensitivity in IBS. Interventional trials assessing tailored dietary modifications, such as combined low-lactose and low-FODMAP approaches, may help define optimal, subtype-specific management strategies. Such integrated, precision-based approaches hold promise for improving therapeutic outcomes and quality of life in patients with IBS across diverse populations.

## Conclusions

This study demonstrates a substantial overlap between LI and IBS in an Indian cohort, with lactose malabsorption identified in 38% of patients. The association was strongest in the IBS-D subtype, indicating that LI is closely linked with symptom patterns in this subgroup; however, the cross-sectional design limits conclusions regarding symptom persistence or causality. These findings underscore that carbohydrate malabsorption is not a coincidental finding but an important factor influencing gastrointestinal symptom patterns, particularly in regions with high lactase non-persistence. By objectively linking dietary lactose exposure, clinical features, and HBT results, this study provides population-specific insight into a key dietary determinant of IBS symptomatology. 

Clinically, the results advocate for integrating dietary history assessment and selective lactose testing into the diagnostic evaluation of IBS, particularly in IBS-D and IBS-M subtypes. Recognizing and managing LI can reduce unnecessary pharmacologic interventions and improve quality of life through simple, individualized dietary adjustments. Future multicenter and longitudinal studies should further explore the interplay between lactose malabsorption, gut microbiota, and visceral hypersensitivity to refine targeted, nutrition-based therapies. The findings from this study contribute essential evidence to refine diagnostic algorithms and optimize therapeutic strategies for IBS. By elucidating the relationship between IBS subtypes and LI, the study supports more precise, individualized management approaches that may substantially improve patient outcomes and quality of life. Collectively, these findings advance a more personalized and diet-responsive framework for managing functional gastrointestinal disorders. 

## Appendices

Appendix A

Diagnostic Reference

The Rome IV diagnostic framework [29] was cited and applied for clinical classification. No tables or questionnaire items from the copyrighted source are reproduced.

## References

[1] Pimentel M, Talley NJ, Quigley EM, Hani A, Sharara A, Mahachai V (2013). Report from the multinational irritable bowel syndrome initiative 2012. Gastroenterology.

[2] Lovell RM, Ford AC (2012). Global prevalence of and risk factors for irritable bowel syndrome: a meta-analysis. Clin Gastroenterol Hepatol.

[3] Ford AC, Moayyedi P, Lacy BE (2014). American College of Gastroenterology monograph on the management of irritable bowel syndrome and chronic idiopathic constipation. Am J Gastroenterol.

[4] Talley NJ, Holtmann G, Walker MM (2015). Therapeutic strategies for functional dyspepsia and irritable bowel syndrome based on pathophysiology. J Gastroenterol.

[5] Longstreth GF, Wolde-Tsadik G (1993). Irritable bowel-type symptoms in HMO examinees. Prevalence, demographics, and clinical correlates. Dig Dis Sci.

[6] Matthews SB, Waud JP, Roberts AG, Campbell AK (2005). Systemic lactose intolerance: a new perspective on an old problem. Postgrad Med J.

[7] Romagnuolo J, Schiller D, Bailey RJ (2002). Using breath tests wisely in a gastroenterology practice: an evidence-based review of indications and pitfalls in interpretation. Am J Gastroenterol.

[8] Farup PG, Monsbakken KW, Vandvik PO (2004). Lactose malabsorption in a population with irritable bowel syndrome: prevalence and symptoms. A case-control study. Scand J Gastroenterol.

[9] Rana SV, Mandal AK, Kochhar R, Katyal R, Singh K (2001). Lactose intolerance in different types of irritable bowel syndrome in North Indians. Tropical Gastroenterology.

[10] Reddy V, Pershad J (1972). Lactase deficiency in Indians. Am J Clin Nutr.

[11] Vesa TH, Seppo LM, Marteau PR, Sahi T, Korpela R (1998). Role of irritable bowel syndrome in subjective lactose intolerance. Am J Clin Nutr.

[12] El-Salhy M (2012). Irritable bowel syndrome: diagnosis and pathogenesis. World J Gastroenterol.

[13] El-Salhy M, Hatlebakk JG, Hausken T (2019). Diet in irritable bowel syndrome (IBS): interaction with gut microbiota and gut hormones. Nutrients.

[14] El-Salhy M, Ostgaard H, Gundersen D, Hatlebakk JG, Hausken T (2012). The role of diet in the pathogenesis and management of irritable bowel syndrome (Review). Int J Mol Med.

[15] Gwee KA, Lu CL, Ghoshal UC (2009). Epidemiology of irritable bowel syndrome in Asia: something old, something new, something borrowed. J Gastroenterol Hepatol.

[16] Hoseini-Asl MK, Amra B (2003). Prevalence of irritable bowel syndrome in Shahrekord, Iran. Indian J Gastroenterol.

[17] Bhatia S (2001). Epidemiology of dyspepsia in Mumbai. Indian Journal.

[18] Ghoshal UC, Abraham P, Bhatt C (2008). Epidemiological and clinical profile of irritable bowel syndrome in India: report of the Indian Society of Gastroenterology Task Force. Indian J Gastroenterol.

[19] Han SH, Lee OY, Bae SC (2006). Prevalence of irritable bowel syndrome in Korea: population-based survey using the Rome II criteria. J Gastroenterol Hepatol.

[20] Kwan AC, Hu WH, Chan YK, Yeung YW, Lai TS, Yuen H (2002). Prevalence of irritable bowel syndrome in Hong Kong. J Gastroenterol Hepatol.

[21] Husain N, Chaudhry IB, Jafri F, Niaz SK, Tomenson B, Creed F (2008). A population-based study of irritable bowel syndrome in a non-Western population. Neurogastroenterol Motil.

[22] Xiong LS, Chen MH, Chen HX, Xu AG, Wang WA, Hu PJ (2004). A population-based epidemiologic study of irritable bowel syndrome in South China: stratified randomized study by cluster sampling. Aliment Pharmacol Ther.

[23] Chang FY, Lu CL, Chen TS (2010). The current prevalence of irritable bowel syndrome in Asia. J Neurogastroenterol Motil.

[24] Celebi S, Acik Y, Deveci SE, Bahcecioglu IH, Ayar A, Demir A, Durukan P (2004). Epidemiological features of irritable bowel syndrome in a Turkish urban society. J Gastroenterol Hepatol.

[25] Masud MA, Hasan M, Azad Khan AK (2001). Irritable bowel syndrome in a rural community in Bangladesh: prevalence, symptoms pattern, and health care seeking behavior. Am J Gastroenterol.

[26] Gonlachanvit S (2010). Are rice and spicy diet good for functional gastrointestinal disorders?. J Neurogastroenterol Motil.

[27] Linlawan S, Patcharatrakul T, Somlaw N, Gonlachanvit S (2019). Effect of rice, wheat, and mung bean ingestion on intestinal gas production and postprandial gastrointestinal symptoms in non-constipation irritable bowel syndrome patients. Nutrients.

[28] Lewis SJ, Heaton KW (1997). Stool form scale as a useful guide to intestinal transit time. Scand J Gastroenterol.

[29] Palsson OS, Whitehead WE, van Tilburg MA (2016). Development and validation of the Rome IV Diagnostic Questionnaire for adults. Gastroenterology.

[30] Szilagyi A (2015). Adaptation to lactose in lactase non persistent people: effects on intolerance and the relationship between dairy food consumption and evaluation of diseases. Nutrients.

[31] Xiong L, Wang Y, Gong X, Chen M (2017). Prevalence of lactose intolerance in patients with diarrhea-predominant irritable bowel syndrome: data from a tertiary center in southern China. J Health Popul Nutr.

